# Increased prevalence of celiac disease in idiopathic inflammatory myopathies

**DOI:** 10.1002/brb3.803

**Published:** 2017-09-05

**Authors:** Olof Danielsson, Björn Lindvall, Claes Hallert, Magnus Vrethem, Charlotte Dahle

**Affiliations:** ^1^ Department of Neurology University Hospital Region of Östergötland Linköping Sweden; ^2^ Department of Clinical and Experimental Medicine Linköping University Linköping Sweden; ^3^ Department of Neurology University Hospital Örebro Örebro Sweden; ^4^ Department of Internal Medicine Linköping University Norrköping Sweden; ^5^ Department of Medical and Health Sciences Linköping University Norrköping Sweden; ^6^ Department of Clinical Neurophysiology University Hospital Region of Östergötland Linköping Sweden; ^7^ Department of Clinical Immunology University Hospital Region of Östergötland Linköping Sweden

**Keywords:** celiac disease, idiopathic inflammatory myopathies, incidence, myositis, prevalence

## Abstract

**Objectives:**

Idiopathic inflammatory myopathies (IIM) are often associated with other immune‐mediated diseases or malignancy. Some studies have reported a high frequency of celiac disease in IIM. The aim of this study was to investigate the prevalence of celiac disease, systemic inflammatory diseases, and malignancy in a cohort of IIM patients, and estimate the incidence of IIM in the county of Östergötland, Sweden.

**Material and Methods:**

We reviewed medical records and analyzed sera from 106 patients, fulfilling pathological criteria of inflammatory myopathy, for the presence of IgA antibodies against endomysium and gliadin. Antibody‐positive patients were offered further investigation with small bowel biopsy or investigation for the presence of antibodies against antitissue transglutaminase (t‐TG). The patients were classified according to Bohan and Peter or Griggs criteria. The presence of celiac disease, systemic inflammatory, and malignant diseases was documented.

**Results:**

Four of 88 patients classified as IIM (4.5%) had biopsy‐confirmed celiac disease, which is higher than the prevalence in the general population, detected with a similar screening procedure (0.53%). Thirty‐three patients (38%) had a systemic inflammatory disease and five (5.7%) a malignancy. The incidence of confirmed IIM in the county of Östergötland was 7.3 per million/year.

**Conclusions:**

The results highlight the high frequency of associated inflammatory and malignant diseases and confirm an increased prevalence of celiac disease in IIM.

## 1 INTRODUCTION

### 1.1 Idiopathic inflammatory myopathies

Idiopathic inflammatory myopathies (IIM) constitute a group of heterogeneous disorders with a presumed autoimmune pathogenesis (Dalakas, 2015). The most important are dermatomyositis (DM), polymyositis (PM), and inclusion body myositis (IBM). The reported incidence and prevalence of IIM have shown great variations, 1.16–19 per million/year and 2.4–33.8 per 100,000, respectively (Meyer et al., 2015). The insights that these diseases often are associated with neoplastic or systemic inflammatory diseases (SID) and affect people in different age groups formed the basis of the Bohan and Peter classification (Bohan & Peter, 1975). Recent knowledge of clinical features, muscle pathology and laboratory findings evoked a need to form new diagnostic entities and definition of subgroups (Arahata & Engel, 1984; Bergua et al., 2016; Griggs et al., 1995), resulting in new diagnostic research criteria, proposed by the European Neuromuscular Centre (ENMC) (Hoogendijk et al., 2004; Rose, 2013). However, classification issues are still debated (Hilton‐Jones, 2011; Lazarou & Guerne, 2013; Troyanov et al., 2014), and in the absence of validating studies, several experts still favor the Bohan and Peter classification (Nagaraju, 2013; Oddis, 2015).

### 1.2 Associated diseases

The notion of IIM associated with SID has in recent years been widened by an increasing number of myositis‐associated autoantibodies (MAA) and myositis‐specific autoantibodies (MSA), detected in blood. These have enabled the distinction of new IIM subgroups, which show differences concerning prognosis and association with neoplastic diseases or SID (Lazarou & Guerne, 2013). The association of IIM to neoplastic disease has been most evident for DM, with an estimated relative risk (RR) of 5.5, compared to an only slightly raised RR of 1.6, for PM (Yang, Lin, Qin, Liang, & Zhong, 2015). The frequency of associated SID has in earlier cohorts, using different definitions, shown great variations, ranging from 15% to 68% (Henriksson & Sandstedt, 1982; van der Meulen et al., 2003; Ng, Ramos, Sultan, & Isenberg, 2009; Troyanov et al., 2005).

### 1.3 Celiac disease

Celiac disease (CD) is an immune‐mediated enteropathy induced by ingestion of gluten in individuals with a genetic susceptibility, mainly conferred by the HLA‐DQ locus (Sollid & Lie, 2005). A population‐based study of CD in northern Sweden found a prevalence of 0.53%, and a study in Denmark supports earlier evidence, indicating a similar prevalence in the Scandinavian countries (Horwitz et al., 2015; Ivarsson et al., 1999; Sjoberg & Eriksson, 1999). More extensive screening methods point to a prevalence in many western countries approaching 1% (Green & Cellier, 2007). The standard investigation for confirming a CD diagnosis is small bowel biopsy, followed by symptom relief after introduction of gluten‐free diet (Green & Cellier, 2007). Signs of malabsorption and gastrointestinal symptoms are classic, but CD may also be clinically silent or associated with autoimmune diseases (Lauret & Rodrigo, 2013). A case of CD with polymyositis that improved after introduction of gluten‐free diet was reported by Henriksson, Hallert, and Walan (1976). The same group later reported five cases of CD in a cohort of 119 patients with IIM, and the need for serological CD screening in this patient group was discussed (Henriksson, Hallert, Norrby, & Walan, 1982). An association of CD with IIM has, however, only found some support in one systematic study (Selva‐O'Callaghan et al., 2007).

The aim of our study was to screen for celiac disease in consecutive patients, diagnosed with IIM, from the region earlier studied by Henriksson et al. (1982), document associated systemic inflammatory diseases and malignancy, and investigate the incidence of IIM in the county of Östergötland, Sweden.

## 2 MATERIAL AND METHODS

### 2.1 Screening of biopsies and inclusion of patients

We searched all muscle biopsy results coded as inflammatory myopathy, that is, showing inflammatory infiltrates and signs of muscle fiber degeneration, during the years 1997–2002 in the register of the *Neuromuscular unit,* Linköping, Sweden. Identifying 99 potentially eligible cases, the inclusion was extended to patients diagnosed 1995–1996. We sent a letter to identified patients, asking for consent to review their medical records and for blood sampling, to screen for antibodies against endomysium (IgA‐EMA) and gliadin (IgA‐AGA). In cases with positive serology, small bowel biopsy was offered. Of 127 eligible patients, 106 (83%) agreed to participate. Most patients (muscle biopsied 1997–2001) were also included in an earlier study, focusing on classification and the diagnostic gain of serial sectioning for diagnosing IIM (Danielsson, Lindvall, Gati, & Ernerudh, 2013). Four patients lacking gastrointestinal symptoms, whose sera showed raised levels of antibodies to AGA, declined a small bowel biopsy, and were, as an alternative, offered to be investigated for the presence of antibodies against antitissue transglutaminase (t‐TG), to increase the specificity for CD (Sugai et al., 2006). All sera were screened for IgA deficiency (i.e*.,* serum IgA <0.07 g/L).

### 2.2 Antibodies

#### 2.2.1 Endomysium antibodies

IgA‐EMA was assessed by indirect immunofluorescence microscopy using fixed sections of monkey esophagus (Bio Systems S.A, Barcelona, Spain) and fluorescein‐isothiocyanate conjugated rabbit antihuman IgA antibodies (DAKO A/S, Glostrup, Denmark). Visible reaction at a serum dilution of 1/10 (titer 10) was considered positive.

#### 2.2.2 Antigliadin antibodies

AGA of IgA class was determined by ELISA, as previously described (Grodzinsky, Hed, Lieden, Sjogren, & Strom, 1990). The cut‐off levels used for IgA‐AGA (40 U/ml) were based on investigations of healthy blood donors (*n* = 1,866) and corresponded to the 97.5th percentile.

#### 2.2.3 Anti‐tissue Transglutaminase

IgA antibodies against tTG were measured by ELISA, using human recombinant tTG (Biosystems Electrabox, Barcelona, Spain), in accordance with the manufacturer's instructions. For IgA‐tTG, the recommended cut‐off level of ≥10 U/ml was used.

#### 2.2.4 Total IgA

Total IgA in serum was detected by routine turbidimetry.

### 2.3 Data collection

From medical records the presence of autoimmune and malignant disease was documented, as were results of laboratory data and muscle strength testing. Data were collected up to a median of 7.5 years after the diagnostic muscle biopsy. All data supporting the diagnoses of IIM, CD, or a systemic inflammatory disease during this time period were noted, as was the development of malignancy up to 4 years after muscle biopsy. Forty‐five of 106 patients were clinically evaluated at our unit. Added information from the other patients was obtained by communication by letter, e‐mail, or telephone with the patients or their treating physicians.

### 2.4 Biopsy investigation

From the 106 participating patients 168 biopsies were analyzed, using routine histological, histochemical, and immunohistochemical stains. Added biopsies (more than one) had been performed for diagnostic purposes or treatment evaluation. Information from all biopsies was, however, used for classification. To detect sparse pathological findings multilevel sectioning of paraffin‐embedded tissue was performed in all cases, and added biopsy sections and stains needed for classification were performed, as previously described (Danielsson et al., 2013). The IIM classification was carried out by the prime investigator, who also reread all muscle biopsies.

### 2.5 Classification

With three modifications the Bohan and Peter classification was used. Only patients with biopsy findings consistent with inflammatory myopathy were included. IBM patients were classified according to Griggs criteria, and in addition to the associated systemic inflammatory diseases, specifically mentioned in the Bohan and Peter classification, the diagnoses antisynthetase syndrome and mixed connective tissue disease (MCTD) were also included. A diagnosis of systemic inflammatory disease, made by the treating rheumatologist, was accepted and unambiguous documentation for a diagnosis of malignancy was pursued.

### 2.6 Investigation of incidence

Newly diagnosed IIM patients during the years 1997–2001, classified as at least probable IIM (Bohan and Peter) or possible IBM (Griggs), with residence in the county of Östergötland, were separately documented. These patients were, as a comparison, also classified according to Amato/ENMC. Patients not participating in the screening for CD were asked for consent, allowing review of their medical records for classification, when needed.

### 2.7 Statistics

Using the general population prevalence of celiac disease in Sweden (0.53%) found by Ivarsson et al. (1999), a power calculation based on one sample proportion Wald z test resulted in the need to include 111 cases to detect a prevalence of 5.0%, and 88 cases for 6.0%, for a modest power of 70% (α = 0.05), in a selected population. Software from *Statistica v 12* (StatSoft InC., Tulsa, USA) was used. For calculating the confidence interval for the prevalence of celiac disease in patients with IIM, the modified Wald method was used, as suggested by Agresti and Coull (Agresti & Coull, 1998). The method allows computation by hand.

### 2.8 Ethical approval

All included patients gave their written informed consent and the study was approved by the regional ethical committee (ref. no. 01‐374).

## 3 RESULTS

### 3.1 Autoantibodies and celiac disease

Three of 106 patients, of them 88 were classified as IIM, were positive for IgA‐EMA and seven for IgA‐AGA (four patients had slightly (40–45) and three markedly (>200) elevated levels). None of the patients was positive in both tests and no patient was diagnosed with IgA deficiency. The first of the three patients with positive IgA‐EMA (titer 160), with clinical features suggesting an antisynthetase syndrome (although Jo‐1 negative), had moderate gastrointestinal symptoms and a duodenal biopsy showing typical histological findings of CD. After introduction of gluten‐free diet, the bowel symptoms and general well‐being improved, but the muscular and respiratory symptoms did not (associated interstitial lung disease). The second patient (EMA titer 80), diagnosed with polymyositis, reported long‐term diarrhea and stomach pain, but declined to have a small bowel biopsy taken or IgA‐tTG antibodies tested. Nevertheless, he chose to start on gluten‐free diet and his gastrointestinal symptoms improved markedly, as did his general well‐being. He experienced no improvement regarding muscle symptoms, however. This patient was judged to have probable CD. The third patient with positive IgA‐EMA (titer 40) had no gastrointestinal symptoms, a normal small bowel biopsy and was negative for IgA‐tTG antibodies, and was hence not diagnosed with CD.

None of the seven patients with positive IgA‐AGA had gastrointestinal symptoms. Three underwent duodenal biopsy, and all had a normal mucosa, the remaining four, who declined a biopsy, were all negative for IgA‐tTG antibodies. Thus, none of the patients with positive IgA‐AGA was diagnosed with CD. Another three patients turned out to have an already diagnosed CD. The diagnosis had in all cases been verified by small bowel biopsy and clinical improvement of gastrointestinal symptoms after introduction of gluten‐free diet (7, 11, and 26 years earlier, respectively), when they were included in the study. These patients, maintaining appropriate diet, were negative in the IgA‐EMA and IgA‐AGA tests. They had all developed definite IBM, and one was also diagnosed with Sjögren syndrome. Thus, four of 88 IIM patients (4.5%) had definite CD, which is higher than the 0.53% prevalence found in the general population screening (CI 1.4%–11%), and the presently estimated population prevalence approaching 1% (Green & Cellier, 2007; Ivarsson et al., 1999). Compiled data with respect to clinical, laboratory, and duodenal biopsy findings from patients with positive serology (EMA or AGA) and the patients with an earlier diagnosed CD are shown in Table 1.

**Table 1:** Data of patients with raised antibody titers against endomysium (EMA‐Abs) or gliadin (AGA–Abs) and of patients with an earlier diagnosed celiac disease

Case	IgA‐EMA	IgA‐AGA	IgA‐tTG	Gastroint. symptoms	Duodenal biopsy	Celiac disease	Bohan and Peter or Griggs
1	1:160	Neg	—a	Yes	Positive^b^	Definite	Definite IIM overlap
2	Neg	>200	—	No	Normal	No	Probable IIM overlap
3	Neg	42	—	No	Normal	No	Not IIM
4	Neg	43	Neg	No	—	No	Definite IBM
5	Neg	Neg	—	Yes	Positive	Definite	Definite IBM
6	1:40	Neg	Neg	No	Normal	No	Probable IIM overlap
7	1:80	Neg	—	Yes	—	Probable	Possible Polymyositis
8	Neg	45	Neg	No	—	No	Possible Polymyositis
9	Neg	Neg	—	Yes	Positive	Definite	Definite IBM
10	Neg	42	—	No	Normal	No	Probable IIM overlap
11	Neg	>200	Neg	No	—	No	Possible Polymyositis
12	Neg	>200	Neg	No	—	No	Not IIM
13	Neg	Neg	—	Yes	Positive	Definite	Definite IBM

^a^ — = not performed. ^b^Positive = biopsy findings consistent with celiac disease.

IgA‐tTG, antitissue transglutaminase antibodies, Neg, not elevated antibodies.

### 3.2 Disease groups

**Figure 1:**
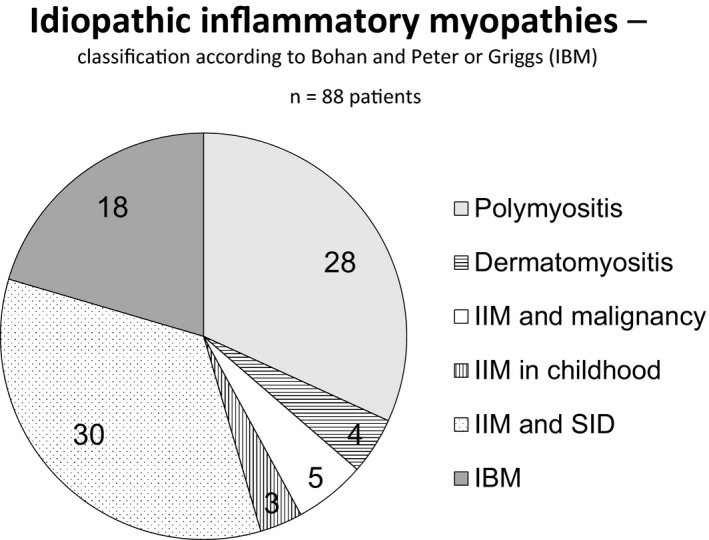
The number of IIM patients in the respective diagnostic group is shown

Eighty‐eight of the 106 included patients (83%) fulfilled the Bohan and Peter or Griggs criteria of at least possible IIM or possible IBM. Of these 88, 28 patients were diagnosed with “isolated” polymyositis, four with “isolated” dermatomyositis, five had an associated malignancy, three were diagnosed with IIM in childhood, and 30 had an associated systemic inflammatory disease, and another 18 had IBM (Figure 1). In the five patients with an associated malignancy (three with DM and two with PM), the primary tumor affected the lung, bladder, ovary, breast, and pancreas, respectively, and a causal connection was evident in the three DM cases. Two patients had an earlier diagnosed and treated malignancy (prostate and colon, respectively) that showed no signs of relapse during follow up. Another two patients were diagnosed with a monoclonal gammopathy with undetermined significance (MGUS), but no malignant transformation was observed. Five patients without diagnosed or suspected malignancy had a follow‐up for less than 4 years. One of these patients died of pulmonary embolism after 6 months and two from unknown causes, after 6 months and 2 years, respectively. For two patients reliable data were available only 3 years after IIM diagnosis.

**Figure 2:**
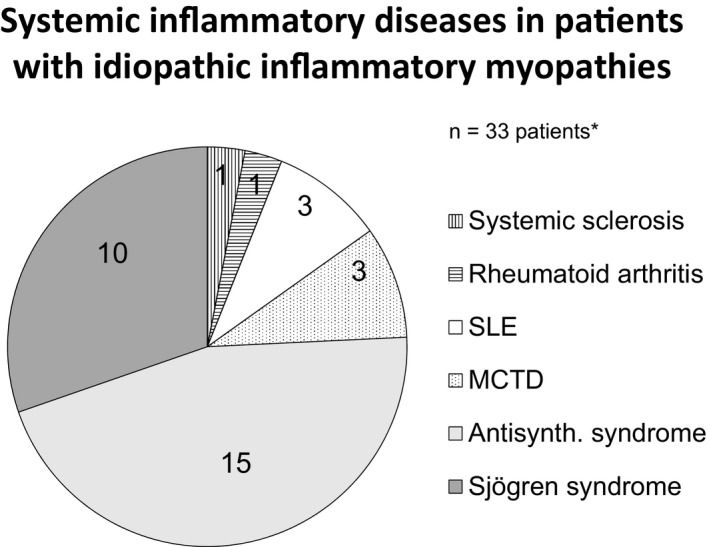
The distribution of patients with systemic inflammatory diseases is shown by the number of cases with the respective diagnosis. *Three of 18 patients with IBM and concomitant Sjögren syndrome are also included. Abbreviation: Antisynth. = Antisynthetase

In the group of 30 patients having an associated inflammatory systemic disease, 15 had an antisynthetase syndrome (Jo‐1 abs were detected in 13), seven Sjögren syndrome, three SLE, three MCTD, one RA, and one systemic sclerosis. In the group diagnosed with IBM, three of 18 patients had concomitant Sjögren syndrome, resulting in a total of 33 IIM patients (38%) with an associated systemic inflammatory disease (Figure 2). Eleven of 22 patients (50%) lacking muscle weakness, also had a systemic inflammatory disease. Data of demography, number of biopsies, and grades of diagnostic certainty in the disease groups are shown in Table 2.

**Table 2:** Characteristics of the study population—demography, follow‐up time, number of biopsies, and grades of diagnostic certainty

	PM *n* = 28	DM *n* = 4	Malignancy *n* = 5	Childhood *n* = 3	Overlap *n* = 30	IBM *n* = 18	Not IIM *n* = 18	Total *n* = 106
Female/male	19/9	2/2	5/0	3/0	24/6	6/12	8/10	67/39
Age^a^ (range)	60 (18–84)	57 (42–60)	60 (47–72)	8 (6.5–16)	48(24–80)	66 (48–76)	51 (26–84)	58 (6.5–84)
Follow up^a^ (range)	7.5 (0.5–18)	9.2 (4.5–18)	5 (0.5–8.0)	13 (12–16)	8.2 (3.0–18)	7.5 (2.5–14)	2 (0.0–20)	7.4 (0.0–20)
Biopsies^b^	49	6	8	4	46	29	26	168
Definite^c^	14	1	4	3	12	16	–	50
Probable^c^	7	2	1	0	14	d	–	24
Possible^c^	7	1	0	0	4	2	–	14

^a^Median years. ^b^1–4 muscle biopsies were taken in each patient. ^c^The Bohan and Peter classification requires two criteria for possible, three for probable, and four for definite diagnosis of IIM. The criteria are as follows: biopsy findings, proximal muscle weakness, EMG findings, raised muscle enzymes, and, in the case of dermatomyositis, a typical skin rash. ^d^The Griggs classification does not have a category for probable IBM.

### 3.3 Incidence of IIM

During the years 1997–2001, biopsies from 29 patients, living in the county of Östergötland with no earlier IIM diagnoses, had findings consistent with an inflammatory muscle disease. Of these, 15 patients (3 per year) met the criteria for probable or definite IIM (Bohan and Peter) or possible or definite IBM (Griggs). Four patients had IBM, four had PM, and one adult patient had DM. There were three patients with childhood DM and three patients had an associated SID. Another four patients were classified as possible IIM. In addition to required biopsy findings, one of these patients had a skin rash typical for DM, and the other three an elevated CK, but none had a proximal muscle weakness. Nine patients had no supportive data for either IIM or any other muscle disease. For one patient data were not considered sufficient for classification. Based on these numbers, the incidence was calculated to 7.3 per million/year. The population average in the county during these years was 412,592 (range 411,333–415,010), source Statistiska Centralbyrån: http://www.scb.se. Fourteen patients fulfilled the criteria according to Amato/ENMC, resulting in an incidence of 6.8 per million/year.

## 4 DISCUSSION

The prevalence of diagnosed CD (4.5%) in this IIM cohort confirms an association with IIM, as earlier indicated by the results by Henriksson et al. (1982). Due to more recent population‐based screening studies, showing a high prevalence rate in the general population (Ivarsson et al., 1999), serological testing is now much more frequently performed, which probably explains why three patients had already been diagnosed with CD at the time of inclusion. Serology screening in this study only identified one new case with definite (biopsy confirmed) and one with probable CD. Both had gastrointestinal symptoms that may nowadays raise a suspicion of CD. Interestingly, three of 18 (17%) patients with IBM in our cohort had CD. Recent studies have also indicated a high prevalence of CD in IBM patients (Badrising et al., 2004; Selva‐O'Callaghan et al., 2007), and both diseases share an association with HLA B8‐DR3 (Badrising et al., 2004). A study specifically addressing systematic screening for CD in IBM may therefore be warranted.

At the start of the study, IgA antibodies against endomysium and gliadin were recommended for CD screening and were used in the prevalence study of Ivarsson et al. (1999), motivating our choice of screening procedure. Since then, IgA‐AGA has been shown to have low sensitivity and specificity for CD, and is no longer recommended as a screening test. The results of this study agree with this conclusion. However, IgA‐EMA as well as IgA‐tTG are regarded as both sensitive and specific markers for CD (Rostom et al., 2005; Sugai et al., 2006), and are presently used as standard screening tests. In contrast to this, Selva‐O'Callaghan et al. (2007) reported biopsy‐confirmed CD in 3 of 17 IIM patients with IgA‐AGA, but none of these patients had raised titers of IgA‐EMA or IgA‐tTG. As possible explanations, the authors suggested that immunosuppressive treatment or the presence of a systemic inflammatory disease may suppress autoantibodies used for screening in this patient group. The sensitivity and specificity of these CD markers in IIM patients thus remain unclear, and negative antibody screening may not be enough to exclude CD in patients with IIM, but should preferably be supplemented by genetic testing or a duodenal biopsy, if suspicion of CD arises in this patient group.

The Bohan and Peter classification has been the most commonly used IIM classification since its publication, often with some modifications (Henriksson & Sandstedt, 1982; Troyanov et al., 2005). The major criticism of this classification has been that it does not require a muscle biopsy and does not include IBM, factors taken into account in this study. In addition, it does not account for the present knowledge of subgroups, identified by clinical features, autoantibodies, and muscle pathology, although the demarcation of these subgroups and their clinical relevance is still controversial. For this study, with a focus on association with systemic inflammatory diseases and malignancy, patients lacking muscle weakness and incidence, the Bohan and Peter classification offered obvious advantages. We further acknowledge the value of the recently proposed classification of IBM (Rose, 2013). But, requiring more specific muscle status data that were not available for this study, its application would not have affected the sensitivity or specificity for the IBM group, as a whole, compared to the used Griggs classification.

Of the 88 patients classified according to Bohan and Peter or Griggs, 33 (38%) had an associated systemic inflammatory disease, and 5 (5.7%) a malignancy. Of note is that 5 of 18 patients (28%) with IBM had either Sjögren syndrome or CD (or both), and among 22 patients lacking muscle weakness 11 (50%) also had a systemic inflammatory disease. This indicates that active search for associated immune‐mediated disorders also is important also in IBM‐patients and IIM patients lacking muscle weakness. In earlier cohorts, disregarding IBM, the found frequencies of an associated malignancy were similar and of an associated systemic inflammatory disease ranging between 15% and 31%, when using similar criteria, but as high as 68%, when widening the definition, considering *overlap features*. (Henriksson & Sandstedt, 1982; van der Meulen et al., 2003; Ng et al., 2009; Troyanov et al., 2005). In our cohort five patients without any raised suspicion of malignancy were followed for less than 4 years, and an undiagnosed neoplastic disease cannot be excluded in these cases. Due to the well‐known risk of misclassifying IBM patients as polymyositis (Rose, 2013), and the relative paucity of inflammation in the more recent, pathologically defined, group, *immune‐mediated necrotizing myopathy* (Hoogendijk et al., 2004) (in this study classified as polymyositis), a particular effort was made to correctly identify and classify these patients.

We found an IIM incidence of 7.3 per million/year in the county of Östergötland, which is in agreement with studies using similar assessments and criteria (Patrick, Buchbinder, Jolley, Dennett, & Buchanan, 1999; Weitoft, 1997), and slightly lower than the estimated incidence (7.98 per million/year) in a recent systematic review (Meyer et al., 2015).

In conclusion, the study confirms an increased prevalence of CD in patients with IIM and suggests liberally screening for CD in this patient group, especially regarding the IBM patients. Associated diseases are common in patients with IIM. In this cohort a systemic inflammatory disease was present in 38% and a malignancy in 5.7%. The detected incidence of IIM in the county of Östergötland, Sweden, was 7.3 per million/year.

## CONFLICT OF INTERESTS

None of the Authors report any conflict of interests.

## References

[brb3803-bib-0001] Agresti, A. , & Coull, B. A. (1998). Approximate is better than “exact” for interval estimation of binominal proportions. The American Statistician, 52(2), 119–126.

[brb3803-bib-0002] Arahata, K. , & Engel, A. G. (1984). Monoclonal antibody analysis of mononuclear cells in myopathies. I: Quantitation of subsets according to diagnosis and sites of accumulation and demonstration and counts of muscle fibers invaded by T cells. Annals of Neurology, 16, 193–208. 6383191 10.1002/ana.410160206

[brb3803-bib-0003] Badrising, U. A. , Schreuder, G. M. , Giphart, M. J. , Geleijns, K. , Verschuuren, J. J. , Wintzen, A. R. , … Dutch, I. B. M. Study Group . (2004). Associations with autoimmune disorders and HLA class I and II antigens in inclusion body myositis. Neurology, 63, 2396–2398. 15623710 10.1212/01.wnl.0000148588.15052.4c

[brb3803-bib-0004] Bergua, C. , Chiavelli, H. , Simon, J. P. , Boyer, O. , Jouen, F. , Stenzel, W. , & Martinet, J. (2016). Immune‐mediated necrotizing myopathy. Zeitschrift fur Rheumatologie, 75, 151–156. 26783154 10.1007/s00393-015-0029-3

[brb3803-bib-0005] Bohan, A. , & Peter, J. B. (1975). Polymyositis and dermatomyositis (first of two parts). The New England Journal of Medicine, 292, 344–347. 1090839 10.1056/NEJM197502132920706

[brb3803-bib-0006] Dalakas, M. C. (2015). Inflammatory muscle diseases. The New England Journal of Medicine, 372, 1734–1747. 25923553 10.1056/NEJMra1402225

[brb3803-bib-0007] Danielsson, O. , Lindvall, B. , Gati, I. , & Ernerudh, J. (2013). Classification and diagnostic investigation in inflammatory myopathies: A study of 99 patients. The Journal of Rheumatology, 40, 1173–1182. 23637317 10.3899/jrheum.120804

[brb3803-bib-0008] Green, P. H. , & Cellier, C. (2007). Celiac disease. The New England Journal of Medicine, 357, 1731–1743. 17960014 10.1056/NEJMra071600

[brb3803-bib-0009] Griggs, R. C. , Askanas, V. , DiMauro, S. , Engel, A. G. , Karpati, G. , Mendell, J. R. , & Rowland, L. P. (1995). Inclusion body myositis and myopathies. Annals of Neurology, 38, 705–713. 7486861 10.1002/ana.410380504

[brb3803-bib-0010] Grodzinsky, E. , Hed, J. , Lieden, G. , Sjogren, F. , & Strom, M. (1990). Presence of IgA and IgG antigliadin antibodies in healthy adults as measured by micro‐ELISA. Effect of various cutoff levels on specificity and sensitivity when diagnosing coeliac disease. International Archives of Allergy and Applied Immunology, 92, 119–123. 2242925 10.1159/000235201

[brb3803-bib-0011] Henriksson, K. G. , Hallert, C. , Norrby, K. , & Walan, A. (1982). Polymyositis and adult coeliac disease. Acta Neurologica Scandinavica, 65, 301–319. 7102258 10.1111/j.1600-0404.1982.tb03088.x

[brb3803-bib-0012] Henriksson, K. G. , Hallert, C. , & Walan, A. (1976). Letter: Gluten‐sensitive polymyositis and enteropathy. Lancet, 2, 317. 10.1016/s0140-6736(76)90772-8 59890

[brb3803-bib-0013] Henriksson, K. G. , & Sandstedt, P. (1982). Polymyositis–treatment and prognosis. A study of 107 patients. Acta Neurologica Scandinavica, 65, 280–300. 7102257 10.1111/j.1600-0404.1982.tb03087.x

[brb3803-bib-0014] Hilton‐Jones, D. (2011). Observations on the classification of the inflammatory myopathies. Presse Medicale, 40, e199–e208. 10.1016/j.lpm.2010.10.035 21377827

[brb3803-bib-0015] Hoogendijk, J. E. , Amato, A. A. , Lecky, B. R. , Choy, E. H. , Lundberg, I. , Rose, M. R. , … Hughes, R. A. (2004). 119th ENMC international workshop: Trial design in adult idiopathic inflammatory myopathies, with the exception of inclusion body myositis, 10‐12 October 2003, Naarden, The Netherlands. Neuromuscular Disorders: NMD, 14, 337–345. 15099594 10.1016/j.nmd.2004.02.006

[brb3803-bib-0016] Horwitz, A. , Skaaby, T. , Karhus, L. L. , Schwartz, P. , Jorgensen, T. , Rumessen, J. J. , & Linneberg, A. (2015). Screening for celiac disease in Danish adults. Scandinavian Journal of Gastroenterology, 50, 824–831. 25687734 10.3109/00365521.2015.1010571 PMC4487537

[brb3803-bib-0017] Ivarsson, A. , Persson, L. A. , Juto, P. , Peltonen, M. , Suhr, O. , & Hernell, O. (1999). High prevalence of undiagnosed coeliac disease in adults: A Swedish population‐based study. Journal of Internal Medicine, 245, 63–68. 10095818 10.1046/j.1365-2796.1999.00403.x

[brb3803-bib-0018] Lauret, E. , & Rodrigo, L. (2013). Celiac disease and autoimmune‐associated conditions. BioMed Research International, 2013, 127589. 23984314 10.1155/2013/127589 PMC3741914

[brb3803-bib-0019] Lazarou, I. N. , & Guerne, P. A. (2013). Classification, diagnosis, and management of idiopathic inflammatory myopathies. The Journal of Rheumatology, 40, 550–564. 23504386 10.3899/jrheum.120682

[brb3803-bib-0020] van der Meulen, M. F. , Bronner, I. M. , Hoogendijk, J. E. , Burger, H. , van Venrooij, W. J. , Voskuyl, A. E. , … de Visser, M. (2003). Polymyositis: An overdiagnosed entity. Neurology, 61, 316–321. 12913190 10.1212/wnl.61.3.316

[brb3803-bib-0021] Meyer, A. , Meyer, N. , Schaeffer, M. , Gottenberg, J. E. , Geny, B. , & Sibilia, J. (2015). Incidence and prevalence of inflammatory myopathies: A systematic review. Rheumatology, 54, 50–63. 25065005 10.1093/rheumatology/keu289

[brb3803-bib-0022] Nagaraju, K. , & Lundberg, I. E. (2013). Inflammatory diseases and other myopathies In Firestein G. S. (Ed.), Kelley′s textbook of rheumatology (pp. 1404–1430). Philadelphia: Elsevier Saunders.

[brb3803-bib-0023] Ng, K. P. , Ramos, F. , Sultan, S. M. , & Isenberg, D. A. (2009). Concomitant diseases in a cohort of patients with idiopathic myositis during long‐term follow‐up. Clinical Rheumatology, 28, 947–953. 19387765 10.1007/s10067-009-1181-4

[brb3803-bib-0024] Oddis, C. V. , & Ascherman, D. P. (2015). Clinical features, classification and epidemiology of inflammatory muscle disease In Hochberg M. C. et al. (Ed). Rheumatology (pp. 1224–1236). Philadelphia: Elsevier Mosby.

[brb3803-bib-0025] Patrick, M. , Buchbinder, R. , Jolley, D. , Dennett, X. , & Buchanan, R. (1999). Incidence of inflammatory myopathies in Victoria, Australia, and evidence of spatial clustering. The Journal of Rheumatology, 26, 1094–1100. 10332974

[brb3803-bib-0026] Rose, M. R. ; Group EIW (2013). 188th ENMC International Workshop: Inclusion Body Myositis, 2‐4 December 2011, Naarden, The Netherlands. Neuromuscular Disorders: NMD, 23, 1044–1055. 24268584 10.1016/j.nmd.2013.08.007

[brb3803-bib-0027] Rostom, A. , Dube, C. , Cranney, A. , Saloojee, N. , Sy, R. , Garritty, C. , … Moher, D. (2005). The diagnostic accuracy of serologic tests for celiac disease: A systematic review. Gastroenterology, 128, S38–S46. 15825125 10.1053/j.gastro.2005.02.028

[brb3803-bib-0028] Selva‐O'Callaghan, A. , Casellas, F. , de Torres, I. , Palou, E. , Grau‐ Junyent, J. M. , & Vilardell‐Tarres, M. (2007). Celiac disease and antibodies associated with celiac disease in patients with inflammatory myopathy. Muscle and Nerve, 35, 49–54. 16967485 10.1002/mus.20652

[brb3803-bib-0029] Sjoberg, K. , & Eriksson, S. (1999). Regional differences in coeliac disease prevalence in Scandinavia? Scandinavian Journal of Gastroenterology, 34, 41–45. 10048731 10.1080/00365529950172817

[brb3803-bib-0030] Sollid, L. M. , & Lie, B. A. (2005). Celiac disease genetics: Current concepts and practical applications. Clinical Gastroenterology and Hepatology: The Official Clinical Practice Journal of the American Gastroenterological Association, 3, 843–851. 16234020 10.1016/s1542-3565(05)00532-x

[brb3803-bib-0031] Sugai, E. , Vazquez, H. , Nachman, F. , Moreno, M. L. , Mazure, R. , Smecuol, E. , … Bai, J. C. (2006). Accuracy of testing for antibodies to synthetic gliadin‐related peptides in celiac disease. Clinical Gastroenterology and Hepatology: The Official Clinical Practice Journal of the American Gastroenterological Association, 4, 1112–1117. 16860613 10.1016/j.cgh.2006.05.004

[brb3803-bib-0032] Troyanov, Y. , Targoff, I. N. , Payette, M. P. , Raynauld, J. P. , Chartier, S. , Goulet, J. R. , … Senecal, J. L. (2014). Redefining dermatomyositis: A description of new diagnostic criteria that differentiate pure dermatomyositis from overlap myositis with dermatomyositis features. Medicine, 93, 318–332. 25500701 10.1097/MD.0000000000000222 PMC4602434

[brb3803-bib-0033] Troyanov, Y. , Targoff, I. N. , Tremblay, J. L. , Goulet, J. R. , Raymond, Y. , & Senecal, J. L. (2005). Novel classification of idiopathic inflammatory myopathies based on overlap syndrome features and autoantibodies: Analysis of 100 French Canadian patients. Medicine, 84, 231–249. 16010208 10.1097/01.md.0000173991.74008.b0

[brb3803-bib-0034] Weitoft, T. (1997). Occurrence of polymyositis in the county of Gavleborg, Sweden. Scandinavian Journal of Rheumatology, 26, 104–106. 9137324 10.3109/03009749709115827

[brb3803-bib-0035] Yang, Z. , Lin, F. , Qin, B. , Liang, Y. , & Zhong, R. (2015). Polymyositis/dermatomyositis and malignancy risk: A metaanalysis study. The Journal of Rheumatology, 42, 282–291. 25448790 10.3899/jrheum.140566

